# Association of Seat Height and Arm Position on the Five Times Sit-to-Stand Test Times of Stroke Survivors

**DOI:** 10.1155/2013/642362

**Published:** 2013-09-11

**Authors:** Shamay S. M. Ng, Susanna Y. Cheung, Lauren S. W. Lai, Ann S. L. Liu, Selena H. I. Ieong, Shirley S. M. Fong

**Affiliations:** ^1^Department of Rehabilitation Sciences, The Hong Kong Polytechnic University, Hong Kong; ^2^Institute of Human Performance, The Univeristy of Hong Kong, Hong Kong

## Abstract

*Objectives*. To investigate (1) the association of seat height and (2) the association of arm position on the five times sit-to-stand test (FTSTS) times of individuals with stroke. *Design*. A cross-sectional study. *Setting*. University-based rehabilitation centre. *Subjects*. Patients (*n* = 43) with chronic stroke. *Methods*. The times in completing the FTSTS with different seat height (85%, 100%, and 115% knee height) and arm positions (arms across chest, hands on thighs). *Results*. FTSTS times were significantly different between 85% and 100% seat heights, and between the 85% and 115% seat heights in both arm positions. However, there was no significant difference between the FTSTS times with the two arm positions at any seat height tested. *Conclusion*. Seat heights lower than the knee height result in longer FTSTS times, whereas arms positions did not significantly affect the FTSTS times.

## 1. Introduction

The ability to rise from a seated position is essential to maintaining physical independence in daily life; however, difficulty or inability in performing this essential action is common after stroke [1]. Initially, the 10 times sit-to-stand (STS) test was developed as a simple, rapid, and reproducible functional assessment for quantifying lower limb strength [2], but that test was found infeasible for assessing some frail individuals unable to rise 10 times consecutively due to weakness or fatigue [2, 3]. Since then, the modified five times sit-to-stand (FTSTS) test has been used to complement tandem, semi tandem, or side-by-side stands and the time-to-walk-8-feet test in the Short Physical Performance Battery [4] to assess lower limb function. The FTSTS test is now the most widely employed functional test to measure lower limb muscle strength [2, 3, 5–8] and to assess fall risk and disability [9, 10]. It has been applied among different populations including older adults [9–12] and subjects with chronic stroke [13, 14], Parkinson's disease [15], vestibular disorders [16], or musculoskeletal problems [3, 6, 7]. 

The FTSTS test has consistently been proven to be reliable functional tool [6–8, 12, 13, 15, 17–19]. In particular, it shows excellent intrarater reliability (ICC > 0.97) on subjects with chronic stroke [13]. Comparable results have been established with subjects with end-stage renal disease (ICC = 0.98) [8] and the community-dwelling elderly (ICC = 0.64) [20]. Likewise, excellent inter-rater reliability has been established for subjects with chronic stroke (ICC = 0.99) [13], Parkinson's disease (ICC = 0.99) [15], and low back pain (ICC = 1.0) [18] and even the healthy elderly (ICC = 1.0) [19]. In terms of the test-retest reliability of the FTSTS test, moderate to excellent reliability has been reported, particularly for subjects with chronic stroke (ICC = 0.98-0.99) [13] and elderly persons with osteoarthritis (ICC = 0.96) [6]. Relatively good test-retest reliability has also been reported for subjects with Parkinson's disease (ICC = 0.76) [15] and older adults in general (ICC range, 0.64–0.96) [17].

The FTSTS test was initially used to measure lower extremity strength [2, 5]. Bohannon et al. reported a significant negative correlation (−0.48 to −0.57) between knee extension force and FTSTS times among healthy community-dwelling individuals aged 50 to 85 [21]. Likewise, testing subjects with stroke, Ng [14] reported a significant correlation of −0.57 between an index of their lower limb muscle strength and FTSTS results. However, STS performance has further been proven multidimensional, not only related to lower extremity strength. FTSTS results have been shown to correlate moderately to strongly (−0.55 to −0.84) with other balance measures among subjects with chronic stroke [13, 14] or balance disorders [16]. Additionally, exercise endurance also contributes to FTSTS performance, with a reported moderate correlation (−0.60) between FTSTS times and 6-minute walk test results among patients with chronic stroke [14].

Although FTSTS is commonly used as outcome measures in stroke [22] and geriatric rehabilitation [23], the protocol of the FTSTS test is not standardized. The height of the chair originally used by Csuka and McCarty [2] was 44.5 cm from the floor. Some researchers used chair heights of 43 cm [16, 24], 45 cm [25], or 46 cm [26], while others adjusted the seat height to the height of the subject's knee [27, 28], or to a height such that the knee was flexed at 90 to 105 degrees [29]. Although greater knee extensor moment is required in standing up from sitting from lower seat height [30], no study to date investigates effects of seat height on FTSTS times.

In addition, arm position of the subject when performing the FTSTS test is not consistent. In most studies, subjects were instructed to fold or cross their arms across the chest [16, 24, 25, 28]. However, subjects were asked to place their hands at their waist [29] or were only told to stand up without using arms in some studies [26]. Some studies did not even mention the arm position at all [27]. Although arm position affects momentum generated during STS and helps shift the body's center of gravity (CoG) forward and upward more effectively [31]; the effect of arm position on FTSTS test has not also been investigated.

We hypothesized that seat height and arm position of the subject during FTSTS test would significantly associate the FTSTS times of individuals with stroke. Therefore, the objectives of this study were to investigate (1) the association of seat height (85%, 100%, and 115% knee height) and (2) the association of arm position (arms across chest and hands of thigh) on the FTSTS times of individuals with stroke.

## 2. Methods

### 2.1. Participants

A convenience sample of 43 community-dwelling stroke survivors, mean 6.8 years poststroke, was recruited from among the clients of a local rehabilitation organization in Hong Kong (mean age (SD): 60 (5.6) years; 31 men, 12 women) (Table 1). Subjects were recruited if they were over 50 years old, had suffered a single stroke at least 1 year before the start of the study, were able to rise from a chair without any arm support, and had an Abbreviated Mental Test score [32] of 7 or above. Potential subjects were excluded if they had any other comorbid neurological disease (e.g., Parkinson's disease) or an unstable medical condition such as cardiovascular problems that might affect proper assessment.

The subjects were informed about the objectives and procedures of the study and invited to sign a consent form before the experiment. The protocol was approved by the Ethics Committee of the Hong Kong Polytechnic University and conducted according to the principles of the Declaration of Helsinki for human experiments. 

### 2.2. Testing Procedure

 An armless, height-adjustable chair with a seat of 28.5 cm depth and a backrest was used in the testing. The test was performed wearing the subject's usual comfortable footwear. The subject started in a seated position with their back against the backrest of the chair. The verbal instructions were “Please stand up and sit down five times as quickly as possible. Stand up until your knees are fully straightened and lean your back against the backrest when you sit down.” Timing began on the command of the assessor and stopped when the subject's back touched the backrest after the fifth stand. Each subject performed two trials in each condition and the times were averaged.

Before the test, the subject was instructed to sit with the knees in 90° of flexion. The knee height was measured from the lateral knee joint line through the lateral malleolus to the ground. Each subject performed the test under six conditions (see Table 2) in order to analyze the effects of different seat heights and arm positions on the FTSTS test times.

The sequence of the six conditions was randomized by drawing lots. A practice trial was given at the beginning of the test. Each subject completed all six conditions in one session. Upon request, subjects could rest for at least two minutes between trials to prevent fatigue. 

### 2.3. Statistical Analysis

The associations of the three different seat heights were analyzed by one-way repeated measures analysis of variance (ANOVA). Paired *t*-tests were used to examine any differences between the two arm positions. Two-way repeated measures ANOVA was applied to test for any interaction between seat height and arm position in influencing the observed times. Finally, Bonferroni's *post hoc* multiple comparison test was used to test for any significant comparisons between the different seat heights. The analysis employed version 18.0 of the Statistical Package for the Social Science software package (SPSS Inc., Chicago. PASW Statistics for Windows, Version 18.0. Released 2009).

## 3. Results

**Figure 1:**
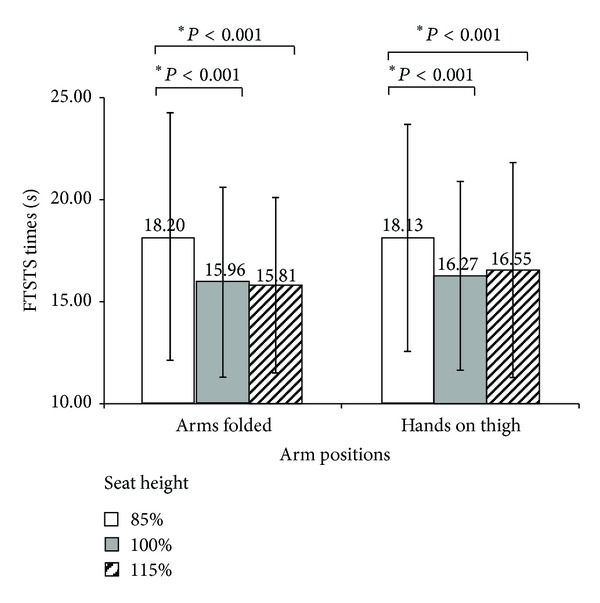
Mean FTSTS times. Seat heights were normalized as a percentage of each subject's knee height. *Indicates difference between knee heights significant at the *P* ≤ 0.05 level of confidence.

The means and standard deviations of the FTSTS times observed in the six conditions are presented in Figure 1. Significant differences were identified between different seat heights with both arm positions. The *post hoc* test revealed that the differences between the 85% and 100% seat heights and between the 85% and 115% seat heights were statistically significant in both arm positions. The differences between the 100% and 115% seat heights were not significant in either arm position. There was no significant difference between the times with the two arm positions at any seat height tested, and there was no significant interaction between seat height and arm position in determining the observed times.

## 4. Discussion

This has been the first study to investigate the association of seat height and arm position on the FTSTS times of stroke survivors. The reported results are consistent with literature reports that lower seat height increases FTSTS test times [33, 34]. The FTSTS times were significantly longer when the seat level was 85% the subject's knee height compared with the 100% and 115% seat heights. 

### 4.1. FTSTS Times after Stroke

The mean times to complete the FTSTS test observed in this study (15.81 to 18.20 s) are consistent with those reported from other studies using subjects with chronic stroke, which range from 13.7 to 19.3 seconds [13, 35–38]. Seat heights in those studies varied from 43 cm [13] or 45 cm [36–38] to 100% of the subject's knee height with the hips flexed at 60° and knees flexed at 75° [35]. Most of the studies adopted an arm-folded position [35, 36, 38]. Two studies required subjects to put their hands on their thighs [13] or simply asked the subjects not to use their upper limbs [37]. The general agreement between these results and those of previous results [13, 35–38] may be explained by recruiting subjects with chronic stroke of similar age.

As expected, the subjects took longer to complete the FTSTS test than healthy elderly persons. The mean FTSTS times of healthy elderly persons aged 57.1 to 71.3 years old have been reported as 7.8 to 12.5 s [13, 21, 23, 35, 39]. The substantial difference should be due in large part to stroke-specific impairments such as muscle weakness [40], impaired sensation [24], and impaired balance [1, 14, 24] following stroke. Loss of motor units [41, 42], reduced firing rates [13, 43], decreased voluntary activation [44], and an increased proportion of fast-twitch fibers [45] in paretic muscles can lead to muscle weakness after stroke, which would be expected to hinder STS performance. Individuals with chronic stroke tend to have weaker knee extensors, which is correlated with less kinetic energy generated during STS maneuvers [1], lengthening the time taken to rise from sitting [1, 24].

Moreover, impaired postural control is common after stroke. It takes longer to stabilize the centre of mass (CoM) and postural sway when standing up and sitting down [27]. Asymmetrical weight bearing on the limbs [1, 40] and somatosensory impairments [24] such as deficits in proprioception and tactile sensation would also be expected to impede STS performance after stroke. 

### 4.2. Seat Height and FTSTS Times

A lower seat brings down the CoM and increases the degree of trunk flexion [31] and the angular displacement of the trunk, hip, knee, and ankle when standing up from sitting [46]. The use of trunk and ankle stabilization strategies should contribute to lengthening the duration of STS transfers [22]. When standing up, initiating lift-off from a lower seat would be more demanding due to the increase in the maximum moment generated by the hips and knees [14]. A lower seat would increase moments at the knees and hips by up to 60% and 50%, respectively, and require more momentum generation and foot repositioning to reduce the muscle strength required of the knee extensors [22]. Lower seat height would also exert greater demands on knee extensors to stabilize the body when moving from sitting to standing [24, 47]. Hence, quadriceps strength is regarded as the most important determinant of the STS times of healthy individuals [13, 24].

 Earlier studies have revealed that muscle strength in the paretic leg [22, 27, 48] and weight-bearing asymmetry [1, 40] are the major factors affecting the STS performance of older individuals after stroke. However, the results of recent studies by Ng [14] suggest that balance ability is a stronger predictor of FTSTS times than muscle strength. When demographic characteristics are controlled for, there is no significant partial correlation between FTSTS times and the muscle strength index, while Berg balance scale scores are a useful independent predictor of FTSTS times [14, 49]. Impaired balance would often increase postural sway during the STS transitions. Lower seat height implies more muscular endurance and efficient postural response to control the CoM [14], so subjects with chronic stroke are likely to take longer to complete the FTSTS sequence when the seat height is lower compared with their healthy counterparts. The results of this study provide evidence that FTSTS times are significantly longer when the seat height is lower. 

### 4.3. Arm Positions and FTSTS Times

There have been few studies looking into the effect of arm position on STS times. The results of this study agree with most of the studies of individuals with stroke or healthy subjects [31, 46–48] that arm positions tested showed no significant relationship with FTSTS times. Jassen et al. [46] have reported finding no significant difference in the time stroke survivors take to rise from sitting with and without arm support. For healthy subjects, Carr and Gentile [47] found that rising with the hands between the knees involved a significantly shorter duration of the maximum support moment (defined as the percentage of the extension phase during which the support moment equals or exceeds 3 times the body weight) compared with rising with the arms restricted. However, the duration of the extension phase (defined as thighs-off to movement end) is similar to the two different arm positions [31]. Etnyre's group [48] has reported similar single STS times with the arms across the chest or the hands on the knees in a study of 100 healthy adults. 

Biomechanical studies of healthy adults have shown that rising from sitting using an armrest results in smaller joint moments at the hips and knees by 50% when compared with rising without an armrest [50]. Thus, using an armrest may require less effort in standing up than rising with the arms restricted. Comparable results have also been reported by Etnyre's group [48], with significantly lower average ground reaction force (GRF) generated with an armrest than with other arm positions. Interestingly, comparing the hands-on-knees and arms-across-the-chest positions tested in this study, Etnyre's group found no significant difference in the average GRF generated [48]. These results indicate that placing the hands on the knees and rising naturally may not make the task easier than rising with the arms across the chest. As few biomechanical studies have examined actual STS times, it remains unclear whether or not the reported kinematic differences between the two arm positions will lead to any difference in STS times. 

 Unexpectedly, the results of this study did not support the idea that arm positions significantly affect FTSTS times. Some authors have reported [51, 52] that pushing with the hands on the thighs is a compensatory strategy commonly used among the elderly [52] and subjects with physical impairment [51]. Mazzà et al. [51] have demonstrated that elderly persons with intermediate functional ability (who scored >13.7 s in the FTSTS test) had to push against their thighs or the chair in order to rise from a seat at 80% or 90% of their knee height. They suggest that pushing against the thighs appears to allow functionally limited elderly persons to overcome the mechanical demands imposed by a low seat height. Mazzà's results did not apply to our study because the subjects were not constrained from pushing on their thighs in the hands-on-knees conditions. In addition, only subjects who could rise from sitting independently without support were studied here, while Mazzà's group recruited subjects with a wider range of functional ability. This may explain why pushing with the hands was not correlated with faster FTSTS times in this study. Most importantly, Mazzà et al. tested only a single STS maneuver, not FTSTS times. 

## 5. Limitations

This study demonstrated a relationship between seat height and FTSTS times, but it did not look into the factors contributing to the observed relationship such as muscle strength [13, 24] and balance [14]. Indeed, no causal relationship has been demonstrated because the study design was cross-sectional. 

Movement quality in performing the sit-to-stand task was not considered in this study, as speed is the main focus of the FTSTS test. There was no restriction on rising with the hands pushing on the thighs in the “hands-on-thighs” conditions. Subjects with poor functional ability might well have pushed themselves up as a compensatory strategy during the tests. In addition, some factors such as foot position [22, 53] and weight-bearing asymmetry [1, 40, 53] which have been shown to affect STS performance were not taken into account in this study. As the population studied was limited to stroke survivors, the results should not be generalized to other disease-specific populations. 

## 6. Future Work

In this study, we studied the association of 3 different seat heights (85%, 100%, and 115% knee heights) and 2 arm positions (arms across chest and hands of thigh) on FTSTS times. Whether the use of other seat height or arm positions would be a more reliable and valid measurement warrants further study. All of our subjects had good level of functional mobility, as all of them were able to rise from a chair without any arm support. Future investigations with larger sample size *s* and subjects with different mobility levels will be warranted.

## 7. Conclusions

 The results show that there is a significant relationship between seat height and FTSTS times, at least among stroke survivors. Seat heights lower than the knee height result in longer FTSTS times. However, the arm positions tested showed no significant relationship with FTSTS times. No optimal seat height for performing the FTSTS test was identified in the study, but clinicians should be aware of the effect of using a low seat when performing the test. Use of a standardized seat height is recommended in order to make FTSTS times comparable among subjects and over time. Further research can evaluate other arm positions and control other variables which may affect FTSTS times. 

## Figures and Tables

**Table 1:** Characteristics of the study population.

Parameters	Number (%) [Range]
Gender	
Male	31 (72.1)
Female	12 (27.9)
Side of Hemiplegia	
Left	20 (46.5)
Right	23 (53.5)
Falls in the past 6 months	
Never	38 (88.4)
More than one fall	5 (11.6)
Present mobility status	
Unaided	22 (51.2)
Cane/Quadripod	21 (48.8)
Age (years)	60.00 ± 5.56 [50–70]
Height (cm)	160.90 ± 6.77 [141–174]
Weight (kg)	66.65 ± 9.46 [50–93]
BMI (kgm^−2^)	25.70 ± 2.88 [20.81–34.58]
Poststroke duration (years)	6.88 ± 2.74 [2.42–16.83]

BMI: body mass index.

**Table 2:** The six conditions performed in the FTSTS test.

Seat heights/arm positions	Arms across chest	Hands on thighs
85% knee height	Condition 1	Condition 2
100% knee height	Condition 3	Condition 4
115% knee height	Condition 5	Condition 6
